# Comparison of fluoride concentration and ph levels in drinking water between rainy and dry seasons in Tláhuac, Mexico City

**DOI:** 10.21142/2523-2754-1403-2026-295

**Published:** 2026-07-10

**Authors:** José Gamarra, Alberto Pierdant-Rodríguez, Nelly Molina-Frechero

**Affiliations:** 1 Maestría en Ciencias Odontológicas, Universidad Autónoma Metropolitana Unidad Xochimilco. Ciudad de México, México. pierdant@correo.xoc.uam.mx, nmolinaf@hotmail.com Universidad Autónoma Metropolitana Maestría en Ciencias Odontológicas Universidad Autónoma Metropolitana Unidad Xochimilco Ciudad de México Mexico pierdant@correo.xoc.uam.mx nmolinaf@hotmail.com

**Keywords:** fluoride, pH, drinking water, drought, fluoruro, disminución de pH, agua potable, estiaje

## Abstract

**Objective:**

To compare seasonal variations in fluoride concentrations and their relationship with pH in drinking water from the Tláhuac borough during rainy and dry periods.

**Materials and Methods:**

A descriptive cross-sectional study was conducted to evaluate fluoride concentrations and pH levels in drinking water during the rainy and dry seasons in the Tláhuac borough. Samples were collected from 53 neighborhoods and stored at 4 °C until analysis. Fluoride concentrations were determined using a potentiometric method with an ion-selective electrode, and pH was measured using a digital pH meter.

**Results:**

A total of 106 drinking water samples from 53 neighborhoods were analyzed across both seasons. The overall mean fluoride concentration was 0.509 ± 0.154 ppm, and the mean pH was 7.25 ± 0.48. Fluoride concentrations were significantly higher during the dry season than during the rainy season (p ≤ 0.0001). Likewise, pH values showed a significant increase during the dry period (p ≤ 0.0002). Pearson correlation analysis revealed a positive association between higher pH values and increased fluoride concentrations (p ≤ 0.049).

**Conclusion:**

Dry-season conditions significantly increased both fluoride concentrations and pH levels in drinking water, highlighting the influence of hydroclimatic and geochemical factors on fluoride mobilization. These findings underscore the need for continuous seasonal monitoring and for considering pH as a key indicator in assessing the risk of fluoride exposure.

## RESUMEN

**Objetivo:**

Comparar las variaciones en las concentraciones de fluoruro y su relación con el pH en el agua de consumo en la alcaldía Tláhuac, durante los periodos de lluvia y sequía.

**Materiales y métodos:**

Se realizó un estudio descriptivo y transversal para comparar las concentraciones de fluoruro y pH en el agua de consumo de la alcaldía Tláhuac durante los periodos de lluvia y sequía. Se recolectaron muestras de 53 colonias, almacenadas a 4 °C hasta su análisis. La cuantificación del fluoruro se efectuó mediante el método potenciométrico con electrodo ion selectivo, y el pH se midió con un medidor digital.

**Resultados:**

Se analizaron 106 muestras de agua provenientes de 53 colonias de la alcaldía Tláhuac durante los periodos de lluvia y sequía. La concentración media total de fluoruro fue de 0.509 ± 0.154 ppm, y el pH promedio de 7.25 ± 0.48. Durante la sequía, las concentraciones de fluoruro fueron significativamente mayores que en la lluvia (p ≤ 0.0001). De igual forma, el pH aumentó de manera significativa en el periodo seco (p ≤ 0.0002). El análisis de correlación de Pearson mostró una relación positiva entre el incremento del pH y las concentraciones de fluoruro (p ≤ 0.049).

**Conclusión:**

La sequía incrementó significativamente los niveles de fluoruro y pH en el agua de consumo, evidenciando la influencia de factores hidroclimáticos y geoquímicos en su movilización. Estos resultados resaltan la necesidad de un monitoreo estacional continuo y de considerar el pH como indicador clave en la evaluación del riesgo por exposición a fluoruro.

## INTRODUCTION

Fluoride is one of the most common inorganic contaminants found in natural and groundwater systems. At elevated concentrations, it constitutes a public health concern due to its association with adverse outcomes such as dental and skeletal fluorosis ^1^. In Mexico, several regions with naturally high fluoride levels have been identified, primarily because of specific geological conditions that favor its presence in the environment ^2^^,^^3^.

Drinking water represents the principal route of fluoride exposure in humans, followed by dietary intake and inhalation. Fluoride in water originates mainly from the dissolution of minerals such as fluorite, fluorapatite, francolite, cryolite, and topaz, which are commonly found in silicic igneous rocks and marine sediments. When these geological formations remain in prolonged contact with water, particularly in groundwater systems, fluoride ions are released into the aqueous phase ^4^^-^^6^.

Fluoride mobilization is influenced by several environmental and physicochemical factors, including climate and water pH. Climatic conditions and groundwater geochemistry are key determinants of fluoride behavior. In regions with high rainfall, frequent aquifer recharge tends to dilute fluoride concentrations as larger volumes of low-fluoride water enter the system. In contrast, arid regions or areas subjected to prolonged drought experience reduced recharge and increased evaporation, leading to the concentration of dissolved ions. Under these conditions, groundwater remains in contact with sediments and fluoride-bearing rocks for longer periods, promoting fluoride dissolution.

Numerous studies have demonstrated marked seasonal variations in fluoride concentrations. For example, research conducted in India reported fluoride levels approximately ten times higher during the dry season than during the rainy season, along with significant correlations with pH, bicarbonate, and other ions, indicating a strong relationship between seasonality and aquifer geohydrochemical conditions. Similarly, Cao et al. ^9^ identified climatic variables as the most robust predictors of groundwater fluoride contamination in China.

Water chemistry, particularly pH, also plays a critical role in fluoride release. Under alkaline conditions, hydroxyl ions (OH-) can displace fluoride ions adsorbed onto minerals such as fluorapatite and fluorinated silicates through ion-exchange mechanisms. Studies on the spatiotemporal variation of aquifer physicochemical parameters have shown that pH may decrease during wet seasons due to rainwater infiltration, CO₂ dissolution, and increased organic matter, which collectively acidify groundwater. Conversely, during dry periods, evaporation and concentration processes favor more alkaline conditions that enhance fluoride release from mineral matrices. Research in granitic terrains has demonstrated that rock weathering consumes CO₂ and increases groundwater pH (pH > 8), thereby facilitating OH-/F- exchange and promoting fluoride mobilization into groundwater. Together, these processes contribute to the dissolution and accumulation of fluoride in aquifers ^7^^-^^12^.

The Tláhuac borough, located in Mexico City, is supplied by a water system that includes groundwater sources and is characterized by marked seasonal variability in rainfall. Despite this, few studies have evaluated seasonal changes in fluoride concentrations and pH levels in drinking water in this area, even though these parameters may significantly influence water quality and population exposure. Therefore, the objective of this study was to compare seasonal variations in fluoride concentrations and their relationship with pH in drinking water from the Tláhuac borough during rainy and dry periods.

## MATERIALS AND METHODS

### Study Design and Ethical Approval

A descriptive cross-sectional study was conducted to compare seasonal variations in fluoride concentrations and their relationship with pH in drinking water from the Tláhuac borough during rainy and dry periods. The study protocol was approved by the Research Commission of the Metropolitan Autonomous University, Xochimilco campus (Approval No. 34504096).

### Study Area and Sample Collection

The study was carried out in the Tláhuac borough, Mexico City. Drinking water samples were collected from 53 neighborhoods distributed across three geographic zones. Samples were collected in 20 mL plastic tubes, labeled according to the zone of collection, and stored at 4 °C until analysis.

Sample collection was performed in two phases: August 2023 and April 2024, corresponding to the rainy and dry seasons, respectively.

All neighborhoods included in the study are located in established urban areas, without rural characteristics or the presence of specific ethnic communities. Although the level of urban infrastructure varies, all neighborhoods are connected to the public drinking water supply system managed by the Mexico City Water System (SACMEX). The neighborhoods share a common distribution network, ensuring a largely uniform water source. However, although the samples originate from the same general system, the water is distributed through pumping stations located at different depths. These variations, together with the geological characteristics of each catchment area, may influence final fluoride concentrations. Consequently, some degree of variability was observed, even among geographically close sampling sites within the same borough.

### Fluoride and pH Analysis

Fluoride concentrations were determined using a potentiometric method with an ion-selective electrode, suitable for quantifying fluoride concentrations ranging from 0.1 to 10 ppm. Measurements were performed using an Orion 4-Star potentiometer (Thermo Electron Corporation). A total ionic strength adjustment buffer (TISAB) solution was added to each sample to control pH, adjust ionic strength, and maintain fluoride ions in solution. Sample storage and analysis were conducted at the laboratory of the Metropolitan Autonomous University, Xochimilco campus.

The analytical method was validated in terms of linearity, repeatability, sensitivity, accuracy, and sample stability. The pH measurements were performed using a GuDoQi wireless digital pH meter. Measurements were taken directly from each sample over a one-minute period, within a measurement range of 0.00 to 14.00 on the pH scale.

### Statistical Analysis

Data was initially recorded in Microsoft Excel and subsequently analyzed using IBM SPSS Statistics version 23. Descriptive statistics were calculated using means and standard deviations. The Mann-Whitney U test was applied to compare fluoride concentrations and pH values between the rainy and dry seasons. In addition, correlation analyses between fluoride concentration and pH were performed, and Pearson’s chi-squared test was used after categorization of continuous variables. For all statistical analyses, a significance level of p ≤ 0.05 was adopted.

## RESULTS

### Fluoride and pH Levels in Drinking Water

A total of 106 drinking water samples from 53 neighborhoods in the Tláhuac borough, distributed across three geographic zones, were analyzed during two sampling periods: August 2023 and April 2024, corresponding to the rainy and dry seasons, respectively.

Across both periods, the overall mean fluoride concentration in drinking water was 0.509 ± 0.154 ppm, with values ranging from 0.200 to 0.934 ppm. The mean pH was 7.25 ± 0.48, with a range of 6.28 to 8.74. The lowest and highest pH values were observed during the dry season (April 2024).

During the rainy season (August 2023), the mean fluoride concentration was 0.394 ± 0.0899 ppm (range: 0.200-0.622 ppm). Similar fluoride levels were observed across the three zones evaluated (Table 1).

**Table 1:** Mean fluoride concentration in drinking water during the rainy season

Area	Mean fluoride (ppm)	Minimum (ppm)	Maximum (ppm)
Northern Area	0.394 ± 0.0906	0.300	0.622
Central Area	0.393 ± 0.0698	0.207	0.520
Southern Area	0.397 ± 0.1080	0.200	0.570

In contrast, during the dry season (April 2024), fluoride concentrations increased compared with the rainy period, reaching a mean value of 0.624 ± 0.114 ppm (range: 0.339-0.934 ppm). The northern zone showed a mean concentration of 0.578 ± 0.0933 ppm (range: 0.417-0.795 ppm), while the central zone exhibited the highest fluoride levels, with a mean of 0.700 ± 0.099 ppm (range: 0.505-0.934 ppm). Notably, Santiago Centro presented the greatest increase in fluoride concentration compared with August 2023. The southern zone showed a mean concentration of 0.592 ± 0.109 ppm (range: 0.339-0.778 ppm), including the lowest fluoride value recorded during this period (Table 2).

**Table 2:** Mean fluoride concentration in drinking water during the dry season

Area	Mean fluoride (ppm)	Minimum (ppm)	Maximum (ppm)
Northern Area	0.578 ± 0.0933	0.417	0.795
Central Area	0.700 ± 0.0998	0.505	0.934
Southern Area	0.592 ± 0.1090	0.339	0.778

### Seasonal Comparison of Fluoride Concentrations and pH Levels

To evaluate the statistical significance of seasonal differences in fluoride concentrations, the Mann-Whitney U test for non-parametric samples was applied. The analysis revealed a statistically significant difference between periods (p ≤ 0.0001), indicating that mean fluoride concentrations were significantly higher during the dry season than during the rainy season.

**Figure 1:**
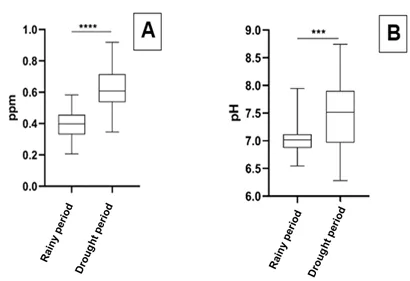
A. Fluoride concentration during the rainy and dry seasons. B. pH levels during the rainy and dry seasons.

A similar analysis conducted for pH values also showed a statistically significant difference between seasons (p ≤ 0.0002), confirming the observed increase in pH during the dry period (Figure 1).

### Relationship Between pH and Fluoride Concentrations

A correlation analysis was conducted between fluoride concentrations and pH values obtained during both sampling periods to assess the association between increased pH and higher fluoride concentrations.

**Figure 2:**
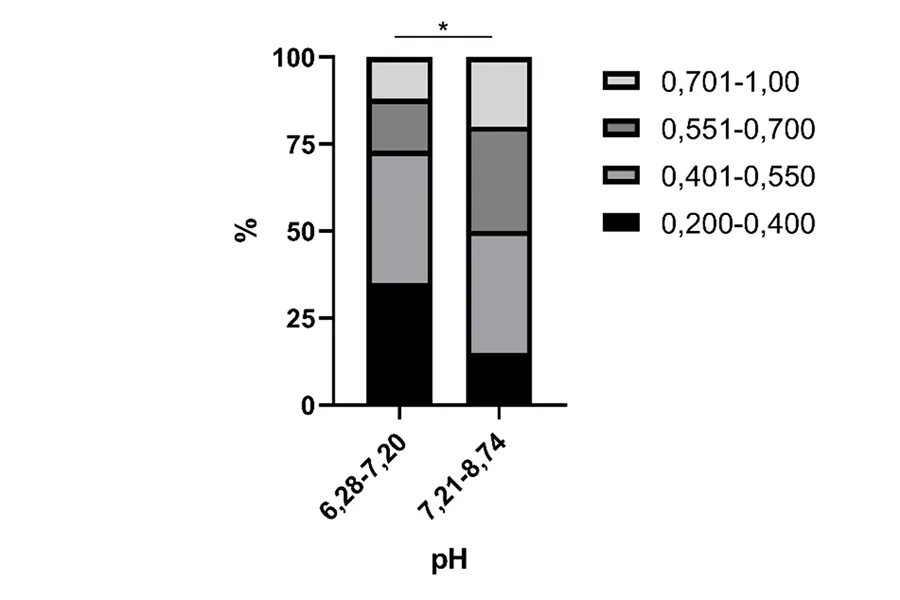
Graph of the correlation between fluoride concentrations and pH levels.

For this purpose, fluoride concentrations were categorized into four ranges: 0.200-0.400, 0.401-0.550, 0.551-0.700, and 0.701-1.00 ppm. pH values were grouped into two categories: 6.28-7.20 and 7.21-8.74 (Table 5). Statistical analysis using Pearson’s chi-squared test yielded a statistically significant association (p ≤ 0.049), indicating that higher fluoride concentrations in drinking water were associated with higher pH values (Figure 2)

## DISCUSSION 

This study is among the few conducted in Mexico that directly examine the relationship between fluoride concentrations and pH variations in groundwater under seasonal climatic conditions. A statistically significant increase in both fluoride concentration and pH was observed during the dry season compared with the rainy season, together with a clear positive correlation between pH and fluoride levels. These findings are consistent with recent studies demonstrating that hydroclimatic factors-such as declining water table levels, higher temperatures, reduced rainfall recharge, and increased evaporation-favor fluoride mobilization in aquifers ^13^. In addition, the dilutive effect of rainfall, which reduces the concentration of dissolved ions, and the limited recharge during drought periods have been widely documented as key mechanisms driving seasonal increases in solute concentrations ^14^.

In Mexico, although seasonally comparative studies remain scarce, national-level evidence confirms the widespread occurrence of elevated fluoride concentrations in arid and volcanic regions ^15^. It has been estimated that more than 20 million people in the country are exposed to drinking water containing fluoride concentrations exceeding 1.5 mg/L ^16^. In the state of Chihuahua, recent studies have reported a substantial proportion of wells with fluoride levels above the maximum permissible limit established by Mexican regulations (NOM-127-SSA1-2021) ^15^.

The results of the present study demonstrate a significant increase in fluoride concentrations during the dry period, accompanied by a parallel increase in pH values. This pattern suggests that aquifer chemical conditions during drought become more favorable for the release of fluoride ions from fluoride-bearing minerals. This behavior is consistent with observations from studies conducted in semi-arid and volcanic regions, where alkaline pH and high alkalinity (HCO₃-) promote fluoride desorption through OH- substitution and enhance the dissolution of minerals such as fluorite and apatite under conditions of reduced recharge and prolonged groundwater residence time ^17^^,^^18^.

Roy et al. ^18^ reported fluoride concentrations ranging from 2.5 to 9.9 mg/L in waters characterized by Ca-Mg-HCO₃- facies in the Eastern Basin of Mexico, which were associated with alkaline conditions and extended drought periods. The authors concluded that groundwater retention and the dissolution of fluorinated minerals were the main processes responsible for these elevated concentrations. Similarly, Ahmad et al. ^19^ identified a strong positive correlation between pH and fluoride (r = 0.72; p < 0.01) in aquifers of southern Punjab, Pakistan, attributing fluoride enrichment to evaporite dissolution, silicate weathering, and cation exchange processes in semi-arid environments. Together, these studies reinforce the hypothesis that alkalinity and reduced rainfall recharge are dominant controls on fluoride mobility in groundwater systems.

In contrast, hydrochemical studies employing managed or controlled aquifer recharge strategies have shown that modifying the pH of infiltrated water alone does not necessarily reduce fluoride release when aquifer sediments exhibit high buffering capacity. However, increasing Ca²+ concentrations has been shown to promote fluorite precipitation, thereby reducing dissolved fluoride levels ^20^. This observation, together with the findings of the present study, highlights that the relationship between fluoride and pH should not be evaluated in isolation, but rather in the context of major ion chemistry and the saturation state of specific subsurface minerals.

At the local scale, the Tláhuac borough is characterized by volcanic geology and soils rich in silicates and carbonates, conditions that favor fluoride leaching during drought periods. The observed increase in pH (7.25 ± 0.48) during the dry season supports the hypothesis that enhanced alkalinity facilitates F-/OH- substitution and desorption from fine mineral phases. Similar mechanisms have been described in recent hydrogeochemical studies of volcanic aquifers in central Mexico ^21^.

To date, only one previous study has compared fluoride concentrations in drinking water during rainy and dry seasons in the Tláhuac borough. Comparison with the study by Hernández-Guerrero et al. ^22^ is particularly relevant, as those authors did not observe significant seasonal differences in fluoride concentrations. This discrepancy may reflect temporal variability in climatic conditions, changes in groundwater extraction patterns, or long-term hydrogeochemical evolution, considering the time elapsed between the two studies.

Taken together, the available evidence suggests that the dominant geochemical processes controlling fluoride release are highly dependent on the hydrological regime and seasonal alkaline conditions within the aquifer. This interpretation is fully consistent with the trends observed in the present study.

The fluoride concentrations observed, particularly during drought periods and seasonal peaks, may have important public health implications if they are widespread or combined with other sources of fluoride exposure. In Mexico, a considerable proportion of urban wells exceed the permissible limit of 1.5 mg/L, increasing the risk of dental and skeletal fluorosis ^16^. In Chihuahua, elevated fluoride concentrations in drinking water and high hazard quotient (HQ) values have been reported, indicating increased non-carcinogenic risk for exposed populations ^17^.

At the global level, probabilistic risk assessment models indicate that in regions affected by fluoride-contaminated groundwater, a significant proportion of the population exhibits non-carcinogenic risk indices greater than one ^16^. In addition, ecotoxicological and epidemiological studies have linked chronic fluoride ingestion to adverse effects on renal, hepatic, and neurological function, particularly in vulnerable populations such as children ^23^.

The seasonal dynamics identified in this study, characterized by higher fluoride concentrations during the dry season, suggest that population exposure is not constant throughout the year but varies temporally. This variability underscores the need to develop time-based monitoring strategies and targeted protection measures during critical exposure periods.

Several limitations should be acknowledged. Because this study was based on measurements obtained during two representative periods (rainy and dry seasons), it does not capture interannual variability or potential multi-year climatic cycles. Long-term monitoring over several years would be necessary to identify trends, fluctuations, and cumulative effects. In addition, increasing the spatial density of sampling points would improve the geographic resolution of fluoride distribution patterns. The inclusion of complementary parameters-such as water temperature, groundwater levels, and geochemical tracers-would also facilitate a more detailed differentiation between recharge, evaporation, ion exchange, and dilution processes ^24^.

Finally, the seasonal patterns observed have relevant clinical implications for dentistry, particularly in the prevention and management of dental fluorosis. Chronic exposure to elevated fluoride concentrations in drinking water during critical periods of tooth development, especially enamel formation, increases the risk and severity of fluorosis, particularly in children under eight years of age ^3^. The higher fluoride concentrations observed during the dry season suggest that actual exposure levels fluctuate throughout the year and may coincide with sensitive stages of dental development. Recent studies assessing ecological and health risks based on seasonal hydrochemical variations further support the conclusion that dry seasons intensify fluoride concentration in aquifers and increase non-carcinogenic health risks ^14^^,^^19^.

These findings reinforce the need for dental professionals and public oral health programs to consider not only annual average fluoride levels, but also seasonal fluctuations when advising patients, recommending the appropriate use of fluoridated products, and assessing individual risk-especially in communities dependent on groundwater supplies.

Future research should focus on the development of seasonal risk maps using geostatistical modeling to identify critical areas of temporary exposure, as well as local epidemiological studies that correlate the prevalence of dental and skeletal fluorosis with long-term hydrochemical records.

## CONCLUSION

The results of this study demonstrate that drought conditions are associated with a significant increase in both fluoride concentrations and pH levels in drinking water, supporting the hypothesis that hydroclimatic and geochemical processes interact to enhance fluoride mobilization. These findings are consistent with the current scientific understanding of the influence of climate, geology, and hydrochemical conditions on fluoride behavior in groundwater systems and have important public health and environmental implications, particularly in vulnerable regions of Mexico.

The results underscore the need for continuous seasonal monitoring of drinking water quality, especially in urban areas supplied by groundwater and characterized by high climatic variability, such as the Tláhuac borough. In addition, they highlight the importance of considering pH as a key hydrochemical indicator when assessing fluoride exposure risk. Timely monitoring and appropriate regulatory measures may help prevent prolonged exposure and contribute to the protection of public health.
